# Optical Efficiency Enhancement of Nanojet-Based Dielectric Double-Material Color Splitters for Image Sensor Applications

**DOI:** 10.3390/nano11113036

**Published:** 2021-11-12

**Authors:** Oksana Shramkova, Valter Drazic, Bobin Varghese, Laurent Blondé, Valerie Allié

**Affiliations:** InterDigital R&D France, Immersive Lab., 975 Avenue des Champs Blancs, 35576 Cesson Sevigne, France; Valter.Drazic@InterDigital.com (V.D.); Bobin.Varghese@InterDigital.com (B.V.); Laurent.Blonde@InterDigital.com (L.B.); Valerie.Allie@InterDigital.com (V.A.)

**Keywords:** image sensor, color splitter, light diffraction, NJ beam deflection, double material element

## Abstract

We propose a new type of color splitter, which guides a selected bandwidth of incident light towards the proper photosensitive area of the image sensor by exploiting the nanojet (NJ) beam phenomenon. Such splitting can be performed as an alternative to filtering out part of the received light on each color subpixel. We propose to split the incoming light thanks to a new type of NJ-based near-field focusing double-material element with an insert. To suppress crosstalk, we use a Deep-Trench Isolation (DTI) structure. We demonstrate that the use of a dielectric insert block allows for reduction in the size of the color splitting element. By changing the position of the DTI, the functionality of separating blue, green and red light can be improved.

## 1. Introduction

Image sensors are solid-state devices which are widely used in consumer electronic devices such as smartphones and various digital cameras. Depending on whether the sensor is a Charge Coupled Device (CCD) or Complementary Metal–Oxide–Semiconductor (CMOS), it will transfer information to the next stage as either a voltage or a digital signal. In the past decades we have observed significant improvement in the performance of CMOS image sensors [1,2,3,4,5,6,7,8,9,10]. Increasing the resolution of these sensors leads to a decrease of the pixel size. As a result of the size scaling each pixel gets a lower amount of light and increased spatial optical crosstalk. To address the existing problems and to improve pixel performance, diverse sensor structures were proposed. It was demonstrated that to increase the light collection efficiency and to improve optical performance, an image sensor with digital-microlenses [11] can be used, with carefully designed periodic surface structures on crystalline silicon [8], or with a front inner lens located on the front side of the backside illuminated pixel [12]. The shape and material of the color filter [13,14,15,16,17,18,19,20,21,22] can also be adjusted. To overcome the electrical and optical crosstalk problems, the Deep-Trench Isolation (DTI) technology was proposed [8,13,16,23,24]. It was demonstrated that, by putting deep trenches filled with lower refractive index materials compared to Si photodiodes between the pixels, it was possible to create a wall against electron diffusion, thus preventing the interference between adjacent pixels.

For decades, an absorptive Bayer color-filter array [25] has remained the dominant approach to achieve color imaging. To discriminate the colors, the color filters are integrated on the pixel array, below the microlenses. It should be noted that by using absorptive color filters in image sensors, about two-thirds of the light entering a pixel is wasted by the filtering process. Some sensor systems, such as the Foveon sensor [26], operate without absorptive color filters or diffractive elements. In such systems, the pixel structure is threefold, with three photodiodes being stacked vertically one on top of the other. The color selectivity principle is that each color band will get absorbed after a different depth in the silicon material. Blue radiation is absorbed in the first photodiode, and red in the last, green being absorbed in between. Potential disadvantages of such systems include the presence of color crosstalk, as the absorption bands somewhat overlap. Moreover, the Foveon sensor pixel size is about 7 μm, which makes it less suitable for smartphone or compact camera formats.

Using color splitters to enable light deflection at different angles for RGB wavelengths, the absorptive losses can be avoided. Recently, several solutions for color splitters based on new topologies were proposed. In order to efficiently separate different spectral components of an optical beam, the use of symmetric and asymmetric deflectors having microscale plate-like structures composed of a transparent medium with refractive index higher than the surrounding material [27] was proposed. This method has several weak points, including a small aperture size of the splitting component, a large aspect ratio between the dimensions of the color splitter element and a high of such device response to the direction of the incoming light. Spatial splitting of incident light into RGB colors for pixel-scale color splitters based on dielectric metasurfaces was demonstrated in [28]. A significant enhancement of the amount of light detected by sensors was observed for metasurfaces with dielectric nanoposts. The dispersion-managed metasurface concept was used to create a RGB color splitter. Low-index nanophotonic color splitters with splitter elements on the surface of CMOS sensors were proposed in [29]. It was demonstrated that the created 2D and 3D structures with as few as four layers efficiency when compared to color filters alone. A perfect color router design is presented in [30]. To achieve color routing for sub-wavelength pixels, the proposed lossless device contains sub-wavelength size scatterers in the region above the pixel’s photodetectors. Due to the multi-scattering process, all incident light for each color channel is routed directly to the photodetector of the corresponding color channel.

Recently, a new type of color splitter was proposed, which separates the light reaching image sensors spectrally and spatially by exploiting the NJ beam phenomenon [31,32,33,34]. A photonic NJ is a highly focused beam of light formed in proximity to the shadow surface of illuminated transparent dielectric particles, with a size comparable to or somewhat larger than the wavelength of the incident optical radiation. It was demonstrated that the focusing properties can be explained by the edge diffraction phenomenon [33]. It was observed that by combining two or more dielectric materials with different refractive indexes, a NJ focusing components could be designed, capable of deviating the focused beam in the near zone [34]. Geometric and material parameters of such a system control the deviation direction and the intensity of the generated NJ beams. The generated beam must be considered as the result of recombination of multiple NJ beams, originating from different edges (associated with different blocks) of the microstructure. We demonstrated that the proposed topologies of multi-material microlenses [35] could help to reduce the size of the color splitting element as well as the optical crosstalk through the dielectric layer.

In this work we propose a new topology of a double material NJ-based color splitter with DTI structures to suppress the crosstalk. The observed color splitting effect relies on light diffraction by the edges of the constitutive parts of the multi-material diffractive element. The NJ beams, originating from different edges of the multi-material elements, recombine and contribute to the formation of a spectral-dependent NJ beam deflection. Using two symmetrically positioned deep-trenches which are located inside the silicon substrate, we can create so-called color channels with modified distribution of the power density. We demonstrate that upon changing the parameters of constitutive parts of the elements and choosing the proper position of deep-trenches, we can obtain mainly red color separation functionality. Taking into account an advantage of the natural light absorbing characteristics of silicon material, we can use two stacked (Foveon-like) photodetectors for separation of blue and green colors transmitted through the same channels. We also can split blue and green putting corresponding photodetectors into the different side channels. To find a proper position of the photodetectors, we analyze the total transmittivity measured at different depths inside the substrate. The proposed solution can improve the quality of filtering and it can increase the sensitivity of a back-illuminated CMOS image sensor.

## 2. Materials and Methods

**Figure 1:**
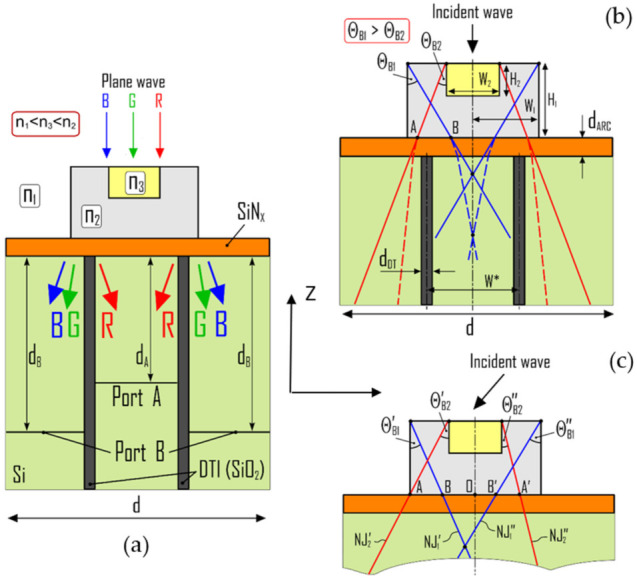
(**a**) Topology of a NJ-based dielectric color splitter for normal incidence; (**b**) Cross-section view of a color splitter with parameters of the system; (**c**) NJ generation by double-material elements for an inclined incidence of a plane wave.

Figure 1a illustrates the general topology of the proposed color splitter. The cross-sectional view of the NJ-element on the top corresponds to a cuboid, cylinder or prism embedded in a homogeneous dielectric host media with a refractive index $n_{1}$ < $n_{2}$. The NJ-element has a dielectric insert block with a refractive index $n_{3}$ < $n_{2}$. We consider the electromagnetic wave incidence from the top of the elements. The topology is arranged in such a way that the NJ beams, originating from external and internal edges and having different intensities and angles of deviation, recombine and contribute to the formation of a spectrally-dependent NJ beam deflection, as illustrated in Figure 1b. The material and size of the insert block with refractive index $n_{3}$ can be selected based in part on the parameters of the outer block. The focal length of the NJ element with insert can be characterized as a function of the size (width or radius) and index ratio of the media inside and outside the microstructure.

Let us present a set of equations to estimate the position of NJs generated by the system with $n_{2}$ > $n_{3}$ > $n_{1}$. The near-field pattern and position of NJ hot spots are determined by the form, size, position of the outer block and the refractive index of the insert block. The effect is the result of an interference of, on one hand, the NJ beams associated with the top edge of the outer block of the NJ elements, and, on the other hand, the NJ beams associated with the top edge of the insert block (see Figure 1b). In this case, the intersection of NJs associated with the edges of different constitutive parts leads to the forming of hot spots located out of the axis of symmetry of the NJ element outer block. The total response of the inhomogeneous systems with dimensions larger than a few wavelengths of an incident wave represents the interplay between the NJ and Fresnel diffraction phenomena.

For the normal incidence of incident wave, the NJ beam radiation angle for constitutive parts of the NJ element can be determined as a function of the ratio between the refractive indexes of the surrounding media and material of the outer block of the lens (for the insert block, the “host medium” is the material of the outer block), and the base angle of the element. For the outer block part of the elements with refractive index $n_{2}$ and vertical edges, the NJ_1_ beam radiation angle can be determined using the following approximate formula:

(1) $$\Theta_{B1}\approx \frac{90^{\circ }-\sin^{-1}\left(\frac{n_{1}}{n_{2}}\right)}{2}$$

The cross point (hot spot) of the two identical and symmetrical NJ_1_ generated by the external edges of the element (outer block) determines the focal length of the single material element. This focal length can be estimated as:

(2) $$F_{L}=\frac{W_{1}}{\tan\Theta_{B1}}$$

where 2$W_{1}$ is the full width of the main part of the NJ element (outer block). Taking the height $H_{1}$ of the outer block approximately equal to the critical height [36] ($h_{c}\approx \lambda /\left(n_{2}-n_{1}\right)$), we can generate the total NJ beam with maximal intensity. To determine the total width of the outer block, we take that $F_{L}$ > $H_{1}$, so we obtain: $W_{1}>\lambda \tan\Theta_{B1}/\left(n_{2}-n_{1}\right)$.

To provide the color splitting functionality we need the input of several NJs with different angles of deviation and different intensity. To generate the NJ_2_, we use an insert with refractive index ${\;n}_{3}$. In the case of a symmetrical inhomogeneous element with an insert for which ${\;n}_{3}$ < $n_{2}$ and $n_{1}$ < $n_{2}$, the two additional similar NJs (NJ_2_) will be generated by the internal edges of the element with the insert. The NJ_2_ beam radiation angle can be determined as:

(3) $$\Theta_{B2}\approx \frac{90^{\circ }-\sin^{-1}\left(\frac{n_{2}}{n_{3}}\right)}{2}$$

The proposed example ratio between the refractive indexes leads to a result in which $\Theta_{B2}<\Theta_{B1}$, and NJ_2_ is less intense than NJ_1_. The size (width and height) of the insert may be selected based on parameters of the outer block and on the refractive index $n_{3}$. If $n_{2}\leq 2$, it is desirable for the generated NJ_2_ not to cross the vertical edges of the main element to avoid the additional NJ refraction at the boundary between the material of outer block and host medium. Thus, parameters may be selected such that AA′ < 2$W_{1}$ and $W_{2}<2\left(W_{1}-H_{1}\tan\Theta_{B2}\right)$. If $n_{2}>2$, NJ_2_ will be reflected by the vertical wall due to the total internal reflection phenomenon. So, to get a maximal distance between NJ_1_ and NJ_2_ in the Si substrate, the width of the insert may be selected to provide favorable conditions to get AA′ as close as possible to the full width of the outer block. The maximal contribution of NJ_1_ may be observed when NJ_1_ does not cross the insert, so we get:

(4) $$H_{2}<\frac{W_{1}-\frac{W_{2}}{2}}{\tan\Theta_{B1}}$$

For the chosen size of the outer block, we will observe at least two NJ hot spots (crossings of NJ_1_ and NJ_2_, see Figure 1b) symmetrically situated relative to the vertical axis of symmetry inside the outer block. Outside the element there may be four NJs penetrating into the Si substrate. First two NJs (NJ_1_) will cross the boundary between the element and substrate at points B and B′. The NJs of second type (NJ_2_) will cross the boundary between the element and substrate at points A and A′. Inside Si, the radiation angles of all these NJs will be reduced due to the refraction phenomenon, and the NJs of different types will be closer to each other.

For better separation of the NJs, we propose to use DTI structures. In an example, two symmetrically positioned (relative to the axis of symmetry of the single element) deep-trenches are located inside the substrate. To get the desirable color splitting function, each deep-trench should be placed between the NJs penetrating into the silicon substrate:

(5) $$W_{1}-H_{1}\tan\Theta_{B1}<\frac{W^{*}}{2}<\frac{W_{2}}{2}+H_{1}\tan\Theta_{B2}\;$$

where $W^{*}$ is the minimal distance between DTs, as shown in Figure 1b.

Let us now consider the effect of the angle of plane wave incidence on the properties of generated NJ beam (see Figure 1c). In the case of plane wave oblique incidence on the outer block with refractive index $n_{2}$ and on the insert, two opposite vertical edges of the corresponding parts will generate two NJs with nonequal beam radiation angles:

(6) $${\Theta ’}_{B1,\;B2}\approx \frac{90{}^{\circ}-\sin^{-1}\left(\frac{n_{1,3}}{n_{2}}\right)}{2}+\frac{\alpha }{2}$$

$$\Theta ’{’}_{B1,\;B2}\approx \frac{90{}^{\circ}-\sin^{-1}\left(\frac{n_{1,3}}{n_{2}}\right)}{2}-\frac{\alpha }{2}$$

where α  is the angle of electromagnetic wave incidence.

A system optimized for normal incidence may have poor splitting functionality in the case of inclined incidence. To improve the efficiency for a wider range of angles of incidence, the parameters of the system must be optimized taking into account that α > 0. Finally, for α > 0 Equation (5) will take the following form:

(7) $$W_{1}-H_{1}\tan{\Theta ’}_{B1}<\frac{W^{*}}{2}<\frac{W_{2}}{2}+H_{1}\tan{\Theta ’’}_{B2}$$

Considering a periodic array of such elements with the inserts, inside the substrate and close to its surface we will observe periodic alternation of the hot spots for the NJs of the same type: NJs of the first type will have their crossing points at the axis of symmetry of the elements; NJs of the second type will provide hot spots at the boundaries of the pitches. Upon changing the pitch of this system, the intensity of the hot spot can be adjusted.

## 3. Results

To evaluate the EM response of the system, numerical simulations of a periodic array of 2D double-material elements with the inserts were performed using the finite element method provided in the commercial COMSOL Multiphysics software (COMSOL Inc., Burlington, MA, USA). It was assumed that the system is illuminated by a linearly TM-polarized wave. To model wave propagation in a single unit cell of the array, on either side of the unit cell we used periodic boundary conditions with Floquet periodicity. To avoid non-physical reflection, we model the open boundaries using perfectly matched layer domains. To measure the changing of the incident light transmittance we scan the power density between the deep-trenches at some depth $d_{A,B}$ inside the Si layer (see Figure 1a).

**Figure 2:**
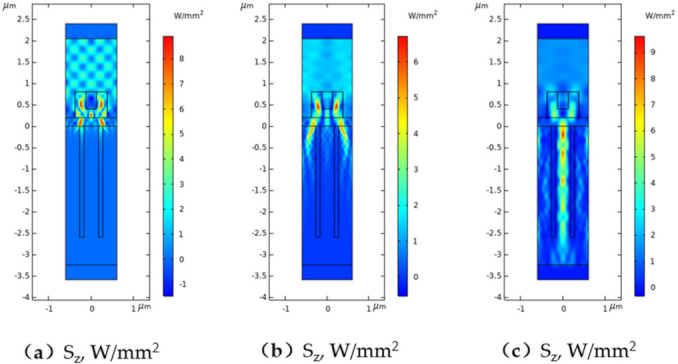
Calculated power distribution in a unit cell with d = 1200 nm, $H_{1}$ = 600 nm, $H_{2}$ = 400 nm, $W_{1}$ = 380 nm, $W_{2}$ = 240 nm, $d_{\mathrm{ARC}}$ = 200 nm, $W^{*}$ = 440 nm, $d_{\mathrm{DT}}$ = 100 nm for (**a**) λ = 400 nm, (**b**) λ = 500 nm, (**c**) λ = 700 nm.

In Figure 2 the color splitting functionality of the proposed system is illustrated using the power distribution for three wavelengths. For the system design, Si_3_N_4_ was used as material for the outer block with refractive index $n_{2}$ (for visible spectrum $n_{2}$ changes from 2.1 to 2.0), MgF_2_ was the material for the insert with refractive index $n_{3}$ (for visible spectrum $n_{3}$ changes from 1.4 to 1.39), SiN_x_ with refractive index 2.04 was used as an antireflection layer, the DTI layers were simulated with SiO_2_ material (for visible spectrum refractive index of SiO_2_ changes from 1.56 to 1.54). It could be seen that for the red color band central wavelength (λ = 700 nm), main power is transmitted through the central channel where we can put Port A (see Figure 1a). In the case of wavelength corresponding to the green color band (centered on λ = 500 nm) the main part of the light is transmitted through the side channels where we can put two Ports B (see Figure 1a). Blue color band (λ = 400 nm) will be also transmitted through the side channels with the smaller depth of power penetration.

**Figure 3:**
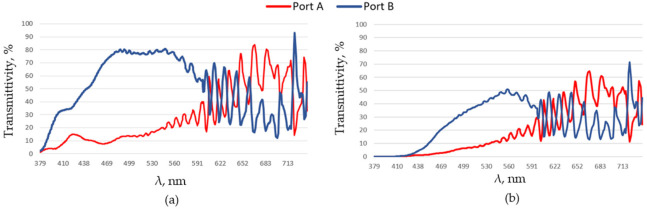
Transmittance for the full visible spectrum at normal incidence and different positions of the ports inside the silicon layer: (**a**) $d_{A}$ = $d_{B}$ = 100 nm; (**b**) $d_{A}$ = $d_{B}$ = 1500 nm.

The dependence of transmitted integral power density on the wavelength for two different positions of the ports (Port A and Port B) inside the Si layer (see Figure 1a) is presented in Figure 3. The simulations presented below correspond to such parameters of the system selected for λ = 620 nm to provide red color splitting functionality. It is assumed that the host medium has a refractive index $n_{1}$ = 1.0, the system is periodic with d = 1200 nm. Using the formula provided above and additional numerical optimization an implementation is selected using a system with $n_{2}$ = 2.0, $n_{3}$ = 1.4, $H_{1}$ = 600 nm, $H_{2}$ = 400 nm, $W_{1}$ = 380 nm, $W_{2}$ = 240 nm. A layer of SiN_x_ with a thickness of $d_{\mathrm{ARC}}$ = 200 nm as an antireflection layer is adopted. The DTI layers are simulated with SiO_2_ material with a refractive index of 1.5; $W^{*\;}$= 440 nm, $d_{\mathrm{DT}}$ = 100 nm. It is possible to observe that at Port A we can register the maximal transmitted power at wavelengths corresponding to the red color, while other wavelengths are registered at Port B.

Our analysis of the effect of anti-reflection coating on the transmittance of the incident light has demonstrated that by increasing the thickness $d_{\mathrm{ARC}}$ we can increase the portion of light transmitted through Port A at the red color wavelengths. Moreover, uniformity of the distribution can be also improved.

**Figure 4:**
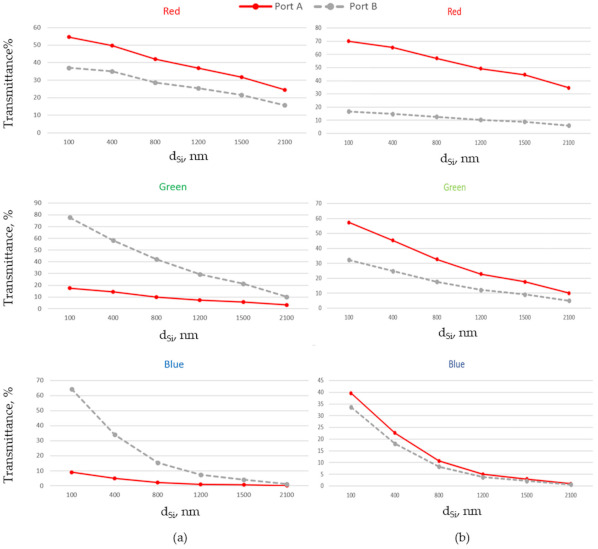
Total transmittance measured for three different RGB colors at normal incidence and $d_{\mathrm{ARC}}$ = 200 nm, $d_{\mathrm{DT}}$ = 100 nm, $W^{*}$ = 440 nm for two different values of refractive index ${\;n}_{3}$: (**a**) $n_{3}$ = 1.4; (**b**) $n_{3}\;$ = $n_{2}\;$ = 2.0.

Figure 4 shows the dependence of total transmittance measured for two ports at different depths $d_{\mathrm{Si}}$, where $d_{\mathrm{Si}}$ corresponds to $d_{A,B}$ inside the Si layer, for three different RGB colors at normal incidence. Based on this dependence we will be able to estimate the penetration depth of the light into silicon material before being absorbed. We can also observe the effect of refractive index of the insert on the color splitting functionalities of the system. It can be seen that for $n_{3}$ = 1.4 (see Figure 4a) Port A effectively registers red colored light. Green and blue colored light can be registered at Port B. Placing the photodiodes or other photodetectors for green and blue colors at different depths we can improve the ability to differentiate between them. Assuming that the threshold for minimal total efficiency transmitted through a corresponding channel is equal to 30%, we can conclude that by placing Port A at distance $d_{A}$ = 100 nm we can effectively register red color with total transmittance corresponding to 55% (see Table 1). To keep transmitted efficiency above the proposed threshold, maximal depth $d_{A}$ of the photodiode should be below 1500 nm. For $d_{B}$ between 700 nm and 1200 nm only green color has total transmittance above 30%. Placing the photodiodes at $d_{B}$ < 700 nm we will detect blue and part of green. Taking into account that blue photons are absorbed near the substrate surface and green are absorbed at some distance [26], we can use two photodiodes (Port B1 and Port B2) in the side channels. Putting the blue photodiode (Port B1 at distance d_B1_) above the green one (Port B2 at distance $d_{B2}$) and close to the surface ($d_{B1}$ = 100 nm, 700 nm < $d_{B2}$ <1200 nm) we will be able to provide blue and green color separation. To avoid the complexity of the stacked photodiodes we can put these detectors into the different side channels.

By increasing the refractive index of the insert, the angles for NJ_2_ are decreased. As a result, the position of point A (see Figure 1b for normal incidence) will be shifted. To observe the effect of an insert on the characteristics of the proposed NJ element, Figure 4b corresponds to the case of single material block with${\;n}_{3}\;$= $n_{2}$. The simulations show that the power transmitted through Port A increases with the refractive index of the insert. Correspondingly, the portion of power for green and blue colors registered at Port B will be decreased. Finally, in the case of a single material block, the main part of the power for the three colors will be transmitted through Port A.

**Figure 5:**
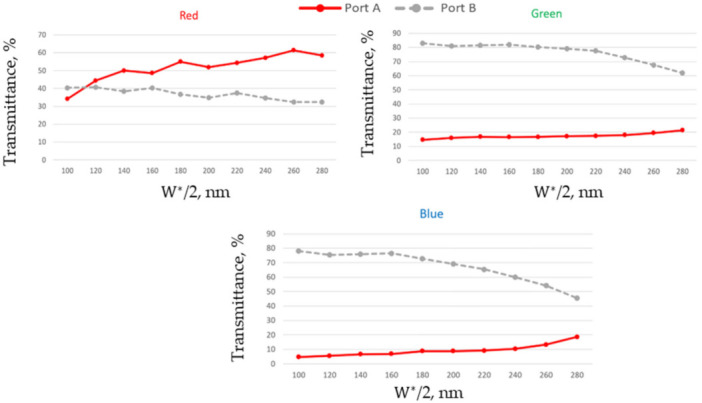
Total transmittance measured for three different RGB colors as a function of the distance between the deep-trenches at normal incidence and $d_{\mathrm{ARC}}$ = 200 nm, $d_{A}$ = $d_{B}$ = 100 nm, $d_{\mathrm{DT}}$ = 100 nm.

The thickness and position of DTIs also affect the color splitting functionality of the device, as shown in Figure 5. The red color high transmittance through Port A can be primarily observed starting from some critical distance between the deep-trenches. The value of the critical distance rises with the thickness of the deep-trenches. It also depends on the position of photodetectors. Increasing $W^{*}$ can significantly reduce the transmittance through Port B corresponding to green and blue colors. The presented simulations were obtained for $d_{A}$ = $d_{B}$ = 100 nm and $d_{\mathrm{DT}}$ = 100 nm. Increasing the DTI width ($d_{\mathrm{DT}}$) leads to an additional decreasing of the green and blue color’s transmittance through Port B. For example, at $W^{*}$ = 440 nm and $d_{\mathrm{DT}}$ = 200 nm we get about 10% less of transmitted green and blue color light at Port B. Increasing the distance $W^{*}$ up to 540 nm for $d_{A}$ = $d_{B}$ = 800 nm we get 50% of transmitted red color light at Port A, 35% of transmitted green color light and less than 10% for blue color at Port B.

**Figure 6:**
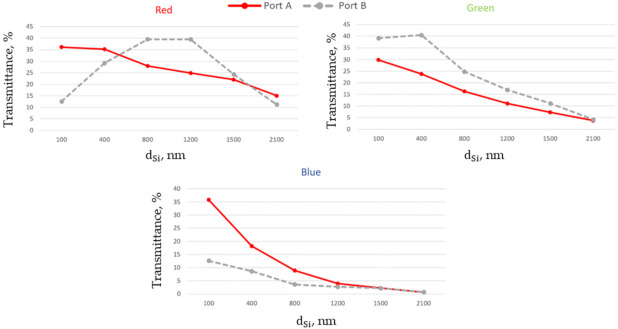
Total transmittance measured for three different RGB colors as a function of the distance between the deep-trenches at normal incidence and $d_{\mathrm{ARC}}$ = 200 nm, $d_{\mathrm{DT}}$ = 100 nm, $W^{*}$ = 440 nm for inclined incidence α = 15°.

The numerical simulations presented in Figure 6 demonstrate the transmittance of RGB colors for an inclined incidence α = 15° at $W^{*}$ = 440 nm. It can be observed that the portion of power transmitted through Port B and corresponding to the blue band dramatically drops with the angle of incidence. At α = 15° the main part of blue color will be transmitted through Port A. In the case of green color, the main part of the power will be transmitted through Port B. We can conclude that to get an effective splitting of red and green colors with blue below the threshold, we must put red and green color detectors at $d_{A}$ = 200 nm–400 nm and $d_{B}$ = 100 nm–400 nm.

Table 2 represents the pixel performance for three different incidence angles and optimal distances $d_{A,B}$ providing red and green color splitting functionality. As the result of data comparison, we obtain that for inclined incidence (5° α 15°) the optimal depths of red and green color detectors are $d_{A}$ = 200 nm–400 nm and $d_{B}$ = 300 nm.

**Figure 7:**
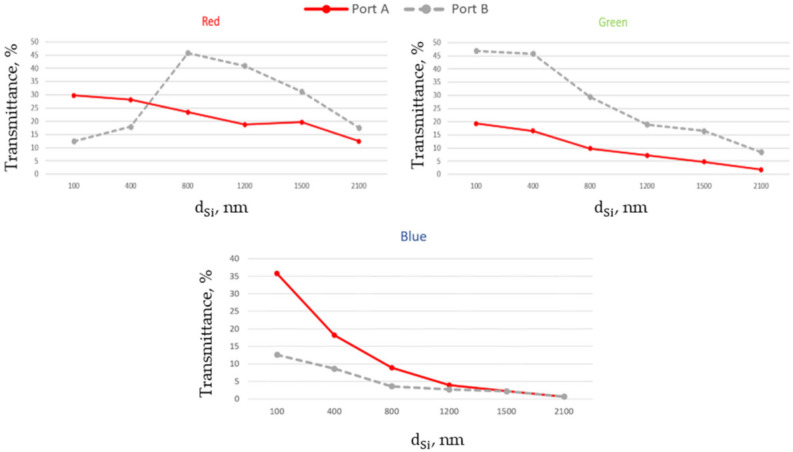
Total transmittance measured for three different RGB colors as a function of the distance between the deep-trenches at normal incidence and $d_{\mathrm{ARC}}$ = 200 nm, $d_{\mathrm{DT}}$ = 100 nm, $W^{*}$ = 360 nm for inclined incidence α = 15°.

As mentioned before, keeping the same parameters for the NJ elements while changing the position of the deep-trench structures can redistribute the portion of power transmitted through the ports due to the limitation of the NJ input. Finally, this dramatically affects the power redistribution in the case of inclined incidence. It was obtained that decreasing $W^{*}$ the green and blue color transmittance through Port B could be improved, but red color transmittance through Port A will be decreased. Figure 7 shows the transmittance of RGB colors for an inclined incidence α = 15° at $W^{*}$ = 360 nm. We can see that placing blue and green color detectors at $d_{A}$ = 100 nm–200 nm and $d_{B}$ = 100 nm–600 nm we can get splitting of blue and green colors. Upon increasing $W^{*}$ the efficiency of the proposed color splitter may be improved for the red color, but blue and green color performance will then become worse.

## 4. Discussion

In this paper we numerically demonstrated a color splitting functionality of a double-material system on a silicon substrate using the NJ phenomenon. Our proposed system is a simple dielectric material block of refractive index ${\;n}_{3}$ inserted in a dielectric outer block of index ${\;n}_{2}$ itself in a host medium of refractive index ${\;n}_{1}$, where ${\;n}_{2}$ > ${\;n}_{3}$ > ${\;n}_{1}$. The position of focal spot, angle of deviation, intensity and shape of NJ beams can be controlled by varying the refractive indexes and sizes of the constitutive parts/blocks. We proposed to combine the double-material elements with DTI structures to suppress the optical crosstalk. Choosing the proper position of deep-trenches, we can enhance the optical efficiency of the proposed color splitter. We demonstrated that upon changing the parameters of constitutive parts of the elements and choosing the proper depth for the detection we can obtain so-called blue, green, and red splitting functionality.

In contrast to the classical absorptive solutions, our proposed double-material system has one or more of the following benefits: better light intake, simpler pixel architecture, relaxed need of a focusing lens on top of the photodiode in the pixel architecture as the color splitter element provides focusing effect as well, reduced risk of crosstalk due to DTI technology, better optical efficiency due to thinner optical stack (less losses) and DTI structure, better angular performance with regards to the angle of the incident light, and the ability to split three colors.

We must note that the proposed solution applies for TE and TM polarizations due to the weak dependence of the angle of deviation of the NJ beam on the incident wave polarization [34]. Noting that the field intensity enhancement in the NJ beam for TE polarization is slightly lower, the system should be optimized taking into account the polarization of an incident wave to get the maximal efficiency.

We must also mention that the fabrication process of the proposed multi-material design is simplified thanks to the lack of high aspect ratios and small feature sizes. Mainly, the complexity of fabrication could be related to the sophisticated shape of the main block. In collaboration with the French company Vmicro (Lille, France), we have fabricated a single-material u-shaped periodic structure as described in [37]. Full u-shape element was fabricated by e-beam lithography using the mask materials for the protection of the different areas at different fabrication steps. To fabricate the multi-material element presented here, we may pattern the new mask and apply the plasma enhanced chemical vapor deposition technique for deposition of the second material.

## Figures and Tables

**Table 1:** Total transmittance measured for three different RGB colors at normal incidence, $d_{\mathrm{ARC}}\;$ = 200 nm, d_DT_ = 100nm, $W^{*\;}$ = 440 nm, $n_{3}\;$ = 1.4.

	Port A, $d_{A}=100\;\mathrm{nm}–1500\;\mathrm{nm}$	Port B, $d_{B\;}=700\;\mathrm{nm}–1200\;\mathrm{nm}$
Blue	10%	30%
Green	20%	45–30%
Red	55–30%	30%

**Table 2:** Total transmittance measured for three different RGB colors at inclined incidence, $d_{\mathrm{ARC}}$ = 200 nm, $d_{\mathrm{DT}}$ = 100 nm, $W^{*}$ = 440 nm and (a) α=5°, (b) α=10°, (c) α=15°.

	$\mathrm{Port}\;A,\;d_{A}=100\;\mathrm{nm}–1500\;\mathrm{nm}$	$\mathrm{Port}\;B,\;d_{B}=300\;\mathrm{nm}$
Blue	15%	30%
Green	20%	62%
Red	50–30%	30%
	**Port A,** $d_{A}$ **= 100 nm–1200 nm**	**Port B,** $d_{B}$ **= 100 nm–400 nm**
Blue	25%	25%
Green	22%	60–50%
Red	45–30%	30%
	**Port A,** $d_{A}$ **= 200 nm–400 nm**	**Port B,** $d_{B}$ **= 100 nm–400 nm**
Blue	30%	15%
Green	30%	40%
Red	35%	30%

## Data Availability

The data presented in this study are available on request from the corresponding author.
